# A Multibranch Object Detection Method for Traffic Scenes

**DOI:** 10.1155/2019/3679203

**Published:** 2019-11-11

**Authors:** Jiangfan Feng, Fanjie Wang, Siqin Feng, Yongrong Peng

**Affiliations:** ^1^Chongqing University of Posts and Telecommunications, Space Big Data Intelligent Technology Chongqing Engineering Research Center, School of Computer Science and Technology, Chongqing 400065, China; ^2^Central South University, School of Computer Science and Technology, Changsha 410000, China

## Abstract

The performance of convolutional neural network- (CNN-) based object detection has achieved incredible success. Howbeit, existing CNN-based algorithms suffer from a problem that small-scale objects are difficult to detect because it may have lost its response when the feature map has reached a certain depth, and it is common that the scale of objects (such as cars, buses, and pedestrians) contained in traffic images and videos varies greatly. In this paper, we present a 32-layer multibranch convolutional neural network named MBNet for fast detecting objects in traffic scenes. Our model utilizes three detection branches, in which feature maps with a size of 16 × 16, 32 × 32, and 64 × 64 are used, respectively, to optimize the detection for large-, medium-, and small-scale objects. By means of a multitask loss function, our model can be trained end-to-end. The experimental results show that our model achieves state-of-the-art performance in terms of precision and recall rate, and the detection speed (up to 33 fps) is fast, which can meet the real-time requirements of industry.

## 1. Introduction

Automatically detecting various objects (such as vehicles and pedestrians) in images or videos from traffic scenes is a basic premise for many intelligent transportation systems. Reasonable traffic management and control based on the movement of vehicles and pedestrians can reduce the occurrence of traffic accidents, road congestion, etc. In this regard, considerable efforts have been made over the past decade. Some challenging benchmarks such as KITTI [1] and LSVH [2] have also been proposed to evaluate and compare the performance of various detection algorithms. Because the generalization of the feature extracted by convolutional neural network is much higher than that of traditional artificial feature, the CNN-based object detection methods have achieved remarkable success on vehicle detection, pedestrian detection, and many other kinds of object detection tasks [3–10].

One of the most popular object detection methods is using sliding windows to generate candidate regions, then features can be extracted from these regions and pretrained classifiers are applied to determine if these regions have certain objects or not. However, it leads to the huge computational cost. Hence, researchers have begun to exploit ways for efficient computation in object detection. Two strategies may be employed: region proposal-based methods and regression-based methods. The former firstly uses region generation algorithms such as selective search (SS) [11] and edge boxes [12] to generate candidate regions (namely, region proposals) and then processes them by convolution neural network, and these methods have high accuracy but cannot meet the requirements of real-time performance. Representative algorithms include RCNN [4], fast RCNN [7], faster RCNN [9], and mask RCNN [13], and they are typical two-stage methods (which generate the proposals using a region generation method and then classify and regress the proposals). The other is the object detection algorithm based on the regression method, which deals with the detection problem as a regression problem and directly predicts the location and classification of the objects. These kinds of methods are typical one-stage methods, and they are fast, but the accuracy is relatively lower than the two-stage methods. Representative algorithms are YOLO [14], SSD [15], YOLOv2 [16], YOLOv3 [17], etc.

**Figure 1:**
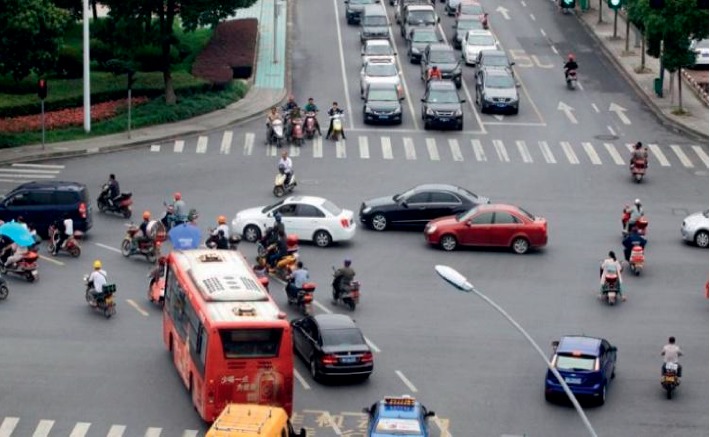
Objects with various scales in traffic scenes.

Despite the powerful performance of CNN, when applying to object detection for traffic scenes, one of the main conundrums is that the traditional CNN-based methods are scale sensitive while it is quite common that the scale of various objects ranges greatly in traffic images or surveillance videos. For example, as shown in Figure 1, the bus has the largest scale and contains far more effective pixels than other objects. Accurately localizing these multiscale instances is quite challenging due to the full connection layer in CNN requires fixed-size input and that the traditional ROI pooling simply replicates some parts of the region proposals to fill the extra space to get feature maps of specified size but after which the original structures of the small objects may have been destroyed nevertheless. In the network training phase, filling in duplicate values will not only lead to inaccurate forward propagation calculation but also cumulate errors in the backward propagation process to impede parameter updating. These two aspects mislead the training of the network and make the network not able to detect small-scale objects accurately. At the same time, small objects may have lost their response when the feature map has reached a certain depth, which undoubtedly makes it more difficult for these methods to detect small objects accurately.

Existing CNN-based studies address the scale-variance problem mainly from two aspects: through the training of different resolution images [18–20] or fusing feature maps with different scales of CNN [5, 8, 10, 21, 22]. Thus, the adaptability of the network in detection tasks with various scales is improved. However, due to the variance of scales, it is difficult to detect objects at all scales because of irrationality on the design of the detection branches or cannot meet the real-time requirement which is essential for unmanned supermarket, autonomous driving, face recognition, parts detection, and many other real-time application scenarios because of the expensive computational overhead caused by too large number of parameters.

**Figure 2:**
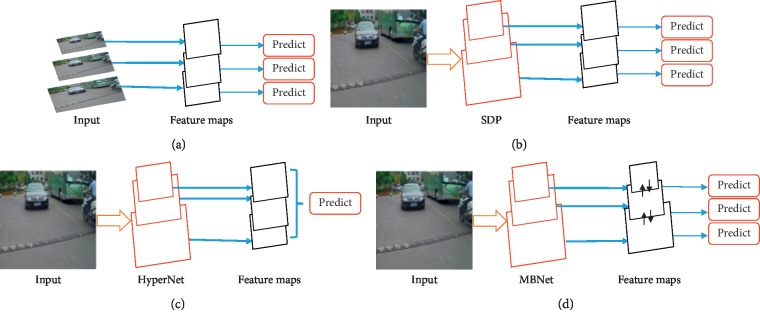
(a) Multiple predictions based on image pyramid. (b) Multiple predictions based on feature pyramid (SDP). (c) Single prediction based on concatenation features (HyperNet). (d) Multiple predictions based on features obtained from multifeature fusion (MBNet).

As suggested by the above discussion, the network architecture for these tasks should consist of multibranches that take in large-, medium-, and small-scale objects, respectively. Recent CNN architectures exploit the property that higher-level features are obtained by composing lower-level ones. Motivated by the idea, we present a multibranch convolutional neural network, named MBNet, to detect multiscale objects in traffic scenes accurately and efficiently. The schematic illustrations of the proposed MBNet and related methods are shown in Figure 2. The MBNet is a regression-based end-to-end network consisting of convolution layer, max pooling layer, upsample layer, route layer, and YOLO detection layer, and specific explanations will be provided in the following sections. Specially, it assigns dynamic weights for the subbranch with respect to the scale of objects and combines multilevel features to detect objects with different scales. Therefore, MBNet can achieve outperforming detection performance in a wide range of input scales and is efficient in terms of computation.

In summary, the main contributions of this paper include:

1. A novel multibranch scale-aware network is proposed for object detection in traffic scenes, incorporating three subnetworks into a unified architecture, which is specialized for the current input scales and boosts the final detection performance with fewer parameters.
2. A scale-aware mechanism is proposed to adjusting the weights accordingly, performing detection accurately for large-, medium-, and small-scale objects from various traffic scenes, which achieves better performance compared with other methods in terms of precision and recall rate and also is able to meet the real-time requirements of application.
3. We construct an urban traffic dataset with large-scale variance, which provides a practical application platform for comparing the performance of various detection algorithms in dealing with different scale objects.

## 2. Related Works

As in other fields, object detection based on traffic scenes has also experienced a period of development, and related tasks include vehicle detection, pedestrian detection, license plate location, and so on. In this paper, we consider a detection method for detecting 7 kinds of objects including pedestrian, car plate, and various vehicles. Early works detect various vehicles using relative motion clues between foreground and background, such as Gaussian mixture model (GMM) [23, 24] and sigma-delta model [25]. They accomplish the task by modeling the distribution of the background as it appears more frequently than the foreground which occupies a small portion of the image. Then, some handcrafted feature-based statistical learning methods which directly detect different objects from images (video frames) have been applied to object detection in traffic scenes. These methods use commonly used features such as HOG, SURF [26], Gabor [27], and Haar-like [28, 29] to describe the image regions, and then pretrained classifiers like SVM, artificial neural network [27], and Adaboost [28] are used to classify the image regions into different categories, such as object area and nonobject area. Aiming at the problem that the existing pedestrian detection algorithms miss detecting in the case of complex scenes or the scale of an object is too small, Chen et al. [30] proposed to cascade simple aggregated channel features (ACF) and rich deep convolutional neural network (DCNN) features for efficient and effective pedestrian detection in complex scenes. In reference [31], a robust license plate location method based on wavelet, transform, and empirical mode decomposition (EMD) analysis is proposed to deal with some challenging problems in practice such as illumination changes and complex background. Some studies combine optical flow with hardware implementation [32] and dense correspondence fields [33] to detect objects. However, these kinds of approaches are unable to distinguish detailed categories of moving objects, such as bicycles, cars, buses, vans, or pedestrians. In addition, these methods also need a slew of complex postprocessing algorithms, such as occlusion recognition and shadow detection, to optimize the detection results.

It is known that traditional CNN-based methods are sensitive to scales, and a lot of subsequent studies have been devoted to addressing this scale-sensitive issue. In reference [2], Hu et al. proposed a new context-aware RoI pooling method to replace the traditional RoI pooling which may destroy the original structure of small objects and further presented a multibranch decision network to conduct the task of box regression and classification. Li et al. [34] proposed to use generative adversarial networks (GANs) to detect small-scale objects and achieved good results. Most of the existing solutions were inspired by two kinds of pyramid representations. One of which applies the concept of image pyramid (Figure 2(a)), which uses input images of multiple sizes to make the network fit for input of all sizes [6,18,19,35]. However, the main drawback of this scheme is that it is computationally heavy, which limits its application in real-time detection. The other conducted by means of feature pyramid, which exploits the information of multiple feature maps extracted from different layers to detect objects with various scales (as shown in Figure 2(b)). The idea of which is to detect small-scale objects with high-resolution shallow features and large-scale objects with low-resolution deep features. This strategy has been adopted in SSD [15], MS-CNN [10], FCN [36], and SDP [5]. However, since the shallow feature maps are the absence of semantic information and the small objects may have lost its response when the feature map has reached a certain depth, the detection effect of these methods on small objects is poor.

In order to make full use of deep layer information to deal with the scale change of the objects, some researchers present to combine feature maps of different layers to train a network (Figure 2(c)), such as HyperNet [37] and MultiPath [22]. However, small objects are still difficult to detect owing to the use of downsampling operations so that small objects cannot maintain ample spatial information when the feature map reaches a certain depth. To take full advantage of the detailed information of shallow features and the semantic information of deep features, another solution is to use high-resolution shallow feature maps and upsampled deep feature maps together to predict small-scale objects, such as [21, 38]. This scheme can better maintain the information of small objects in the deep feature maps, and this is exactly the idea adopted in this paper (Figure 2(d)).

In a word, through the reasonable design and adjustment of the three detection branches, our approach is to achieve performance balance in time, cost, and detection accuracy, which can better detect objects with various scales while meeting the real-time requirements of application. We explore a simple and effective framework that consists of three subnetworks to generate the corresponding detection results on each branch, and then filtering algorithms (such as NMS) are used to refine these results to get the final results.

## 3. MBNet

**Figure 3:**
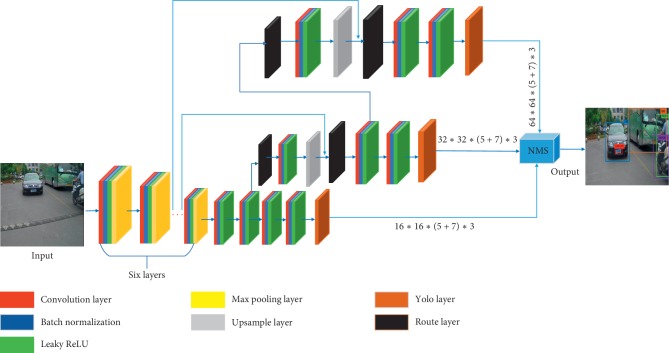
The overall structure of MBNet.

Motivated by the concept of feature pyramid, we propose a new algorithm, i.e., the MBNet. The MBNet is an ensemble of three subnetworks in which scale-specific feature map is employed to detect the objects in traffic scenes of large-, medium-, and small-scale sizes, respectively, as is shown in Figure 3. By fusing the features of different layers, the feature maps used for detection in our model have both rich semantic information of high-level features and detailed information of low-level features, which effectively improves the detection effect of small objects. The design of our model enables MBNet to accurately capture the characteristics of different scale objects on different branches and then classify and locate them. Finally, a series of filtering algorithms are used to screen out the detection results to obtain the final results. The details of the MBNet framework are given in Figure 3.

### 3.1. Predictions across Scales

Drawing on the idea of faster RCNN, we use k-means to cluster the anchor boxes with the help of a series of marked ground truth boxes, which can automatically determine the sizes and number of anchors. Then, 9 clusters have been selected and further extract features through several convolutional layers to produce feature maps specialized for range of object scales. Specially, in the experiments with urban traffic dataset (Section 4.1.1), we predict 3 bounding boxes on the feature map of each detection branch so the tensor is *N* × *N* × (3 × (4 + 1 + 7)) for the 4 bounding box offsets, 1 objectness prediction, and 7 class predictions. As for the three branches designed in the MBNet, *N* stands for 16, 32, and 64, respectively. As the anchor is sensitive not only to detection efficiency but also to localization quality, the method of k-means clustering is used to find the proper *k* value by adjusting the objective function *d*(box, centroid)=1 − IOU(box, centroid) to the minimum as done in YOLOv2 [16], in the function variable, *box* represents the information of the bounding box and *centroid* represents the information of the cluster center, and the appropriate value of *k* after the clustering is 9. The resolution of each image in our handcrafted dataset is 512 × 512, and the 9 clusters on the dataset are (11 × 12), (15 × 30), (43 × 32), (37 × 74), (62 × 87), (69 × 139), (173 × 145), (255 × 278), and (453 × 432).

With semantic information and traffic scene details insufficiently encoded, feature extractor only describes the appearance contents at a coarse level. In order to capture the complementary information, we integrate deep semantic segmentation feature maps into the original object detection framework. In detail, with a series of convolution and max pooling operations, a feature map of a specified size can be learned automatically as the first detection branch. Next, we take the feature map from several layers previously and improve the resolution by a factor of 2, and then we combine it with another feature map as one of the detection branches. We also fetch a lower-level feature map and merge it with another upsampled feature map using concatenation, and several convolution operations are then performed on this combined feature map before it serving as the last detection branch. The combination of low-level descriptors and high-level features could potentially lead to better performances in distinguishing fine-grained categories of objects. In short, MBNet has carefully designed three different detection branches to cover large-, medium-, and small-scale objects as much as possible in traffic scenes.

### 3.2. Network Training Process

Table 1 illustrates the architecture of MBNet in detail. The network consists of 32 layers, including 17 convolution layers for feature extraction, 6 max pooling layers for simplifying feature maps, 2 upsample layers for obtaining high-dimensional feature maps (upsample a layer by improve the resolution by a factor of 2 and then concatenate it to another layer), and 3 yolo layers for receiving output feature maps, which are also serving as three different detection branches in this network. Besides, 4 route layers are used to take a feature map at a certain layer or fuse feature maps from different layers. In the convolution layer, we use regularization to suppress over-fitting and increase the specific gravity of some important parameters in the convolution kernel to extract more accurate feature maps. The batch normalization layer is added after each convolution layer to normalize the data output, which greatly improves the training speed and avoids the occurrence of gradient vanishing. In the network, we use the Leaky ReLU function as the activation function.

**Figure 4:**
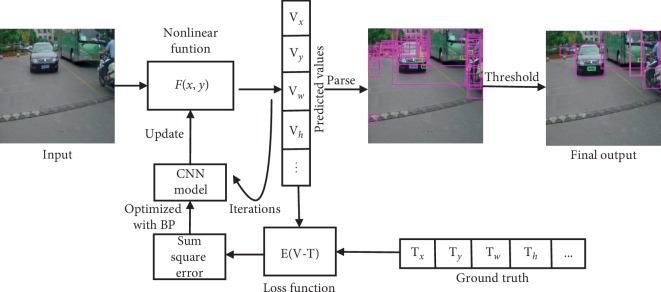
Schematic diagram of network training process.

The network treats the whole detection task as a regression task, dividing the input images into 16 × 16, 32 × 32, and 64 × 64 small regions (grid cells), respectively. Then, each small region (grid cell) predicts three bounding boxes that might contain objects as well as the probability values of each category in this region. Then, we compare these boxes with ground truth and get the error. The whole training process is shown in Figure 4: we treat the trained network as a function containing several parameters, which is abbreviated as *F*(*x*, *y*), where *x* represents input of some dimensions and *y* stands for its output. Firstly, the network is initialized randomly, and then the images in the training set are used as input to get the corresponding output, that is, the bounding box coordinates predictions, objectness prediction, and 7 category predictions, as is shown in Figure 4. As the input of a module can be computed by working backwards from the gradient with respect to the output of that module, the BP algorithm is used to update the parameters in the network to adjust the coefficient values in our function *F*(*x*, *y*) for the next round of training. Then, we iterate in this way until our loss function reaches a certain range or when the number of iterations reaches a certain number of times we terminate the iteration. Next, we choose the loss function value and the most representative network weight value as the final parameters of our network to do the prediction. In the test phase, for each input image, the network produces various scales of output in different detection branches. Next, we combine them together and then use the filtering algorithms such as nonmaximum suppression (NMS) to refine the results.

**Figure 5:**
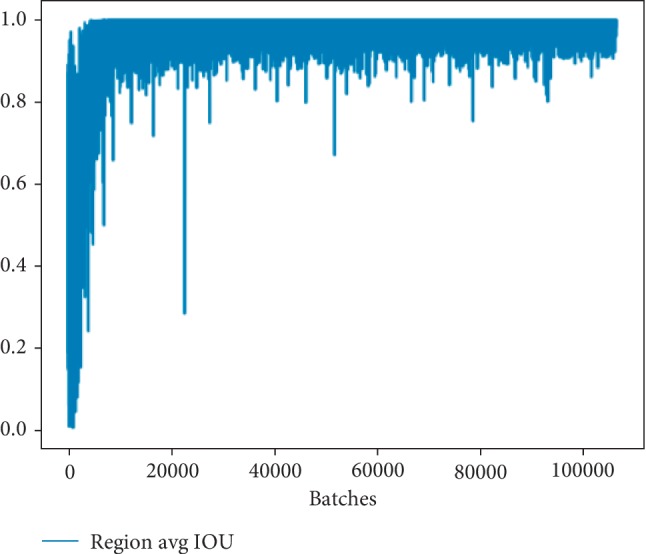
The relationship between average IOU and training times.

**Figure 6:**
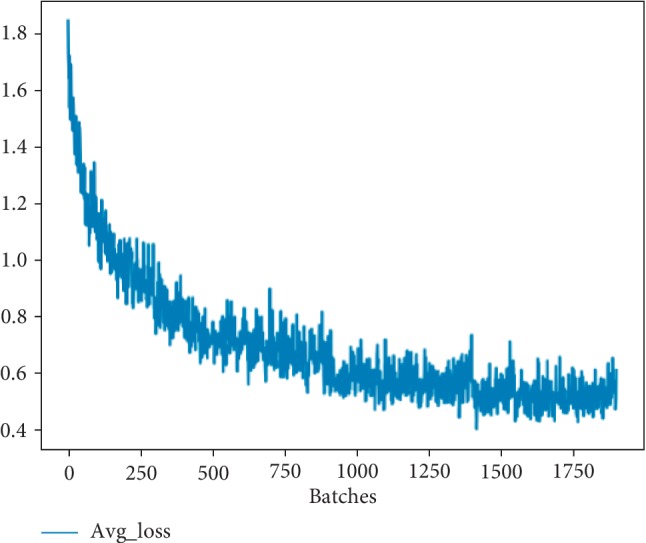
The relationship between loss function and training times.

We have trained the network for 100000 times and obtained the relationship between the average IOU and loss function with the number of training times, as is shown in Figures 5 and 6, respectively. From these figures, we can draw a conclusion that the loss of the network is converging in the iterative process while the value of average IOU is increasing as good as 1.

### 3.3. Bounding Box Prediction and Class Prediction

Following faster RCNN and some other works, our system predicts bounding boxes using dimension clusters such as k-means as anchor boxes prior. When an input image is divided into a *S* × *S* grid, each grid cell predicts B (9 clusters divided up evenly across 3 branches, so here B is 3) bounding boxes under the prerequisite that MBNet predicts three types of anchor boxes in three detection branches (16 × 16, 32 × 32, and 64 × 64). One object is predicted by the grid cell in which the center of the object falls, and the network predicts 4 coordinates for each bounding box, *t*_*x*_, *t*_*y*_, *t*_*w*_, and *t*_*h*_, where (*t*_*x*_, *t*_*y*_) is the offset of the center of ground truth box from the top left corner of the grid cell responsible for the prediction and (*t*_*w*_, *t*_*h*_) is the scale by which the size of a bounding box is zoomed to a size similar to a ground truth box. They are calculated corresponding to

(1) $$\begin{array}{c} t_{x}=G_{x}-C_{x}, \end{array}$$

(2) $$\begin{array}{c} t_{y}=G_{y}-C_{y}, \end{array}$$

(3) $$\begin{array}{c} t_{w}=\log\left(\frac{G_{w}}{P_{w}}\right), \end{array}$$

(4) $$\begin{array}{c} t_{h}=\log\left(\frac{G_{h}}{P_{h}}\right). \end{array}$$

If the cell is offset from the top left corner of the image by (*C*_*x*_, *C*_*y*_) (as is shown in equations (1) and (2)) and the anchor box prior has width *P*_*w*_ and height *P*_*h*_, then the prediction of the coordinates of the predicted bounding box can be obtained by the following equations:

(5) $$\begin{array}{c} b_{x}=\sigma \left(t_{x}\right)+C_{x}, \end{array}$$

(6) $$\begin{array}{c} b_{y}=\sigma \left(t_{y}\right)+C_{y}, \end{array}$$

(7) $$\begin{array}{c} b_{w}=P_{w}e^{tw}, \end{array}$$

(8) $$\begin{array}{c} b_{h}=P_{h}e^{th}, \end{array}$$

where *G*_*x*_, *G*_*y*_, *G*_*w*_, and *G*_*h*_ refer to the center coordinates as well as the width and height of ground truth, respectively. *P*_*w*_ and *P*_*h*_ denote the width and height of the anchor box, respectively. From equations (1)–(8), the 4 predictive output coordinates of the bounding box are obtained. Using *σ*(.) to compress *t*_*x*_ and *t*_*y*_ into [0, 1] region can effectively ensure that the object center is in the grid cell that carries out the prediction and prevent excessive deviation.

During training, we use sum of squared error loss, and the total loss function of our network is shown in equation (9), which is the same as used in YOLOv2 [16]. The design goal of the loss function is to achieve a balance between the coordinates, the confidence of the bounding boxes, and the classes. Our gradient is the ground truth values (calculated from the ground truth box) minus our prediction values, as shown in the fourth and fifth items of the following equation

(9) $$\begin{array}{c} \lambda_{\text{noobj}}\overset{s^{2}}{\underset{i=0}{\sum }}\overset{B}{\underset{j=0}{\sum }}l_{ij}^{\text{noobj}}{\left(c_{i}-{\overset{⌢}{c}}_{i}\right)}^{2}+\lambda_{\text{obj}}\overset{s^{2}}{\underset{i=0}{\sum }}\overset{B}{\underset{j=0}{\sum }}l_{ij}^{\text{obj}}{\left(c_{i}-{\overset{⌢}{c}}_{i}\right)}^{2}+\lambda_{\text{class}}\overset{s^{2}}{\underset{i=0}{\sum }}\overset{B}{\underset{j=0}{\sum }}l_{ij}^{\text{obj}}\underset{c\in \text{classes}}{\sum }{\left(p_{i}\left(c\right)-{\overset{⌢}{p}}_{i}\left(c\right)\right)}^{2}+\lambda_{\text{coord}}\overset{s^{2}}{\underset{i=0}{\sum }}\overset{B}{\underset{j=0}{\sum }}l_{ij}^{\text{obj}}\left(2-w_{i}\;*\;h_{i}\right)\left[{\left(x_{i}-{\overset{⌢}{x}}_{i}\right)}^{2}+{\left(y_{i}-{\overset{⌢}{y}}_{i}\right)}^{2}+\;{\left(w_{i}-{\overset{⌢}{w}}_{i}\right)}^{2}+{\left(h_{i}-{\overset{⌢}{h}}_{i}\right)}^{2}\right]+0.01\;*\;\overset{s^{2}}{\underset{i=0}{\sum }}\overset{B}{\underset{j=0}{\sum }}l_{ij}^{\text{noobj}}\left[{\left(p_{jx}-{\overset{⌢}{x}}_{i}\right)}^{2}+\;{\left(p_{jy}-{\overset{⌢}{y}}_{i}\right)}^{2}+{\left(p_{jw}-{\overset{⌢}{w}}_{i}\right)}^{2}+{\left(p_{jh}-{\overset{⌢}{h}}_{i}\right)}^{2}\right]. \end{array}$$

**Figure 7:**
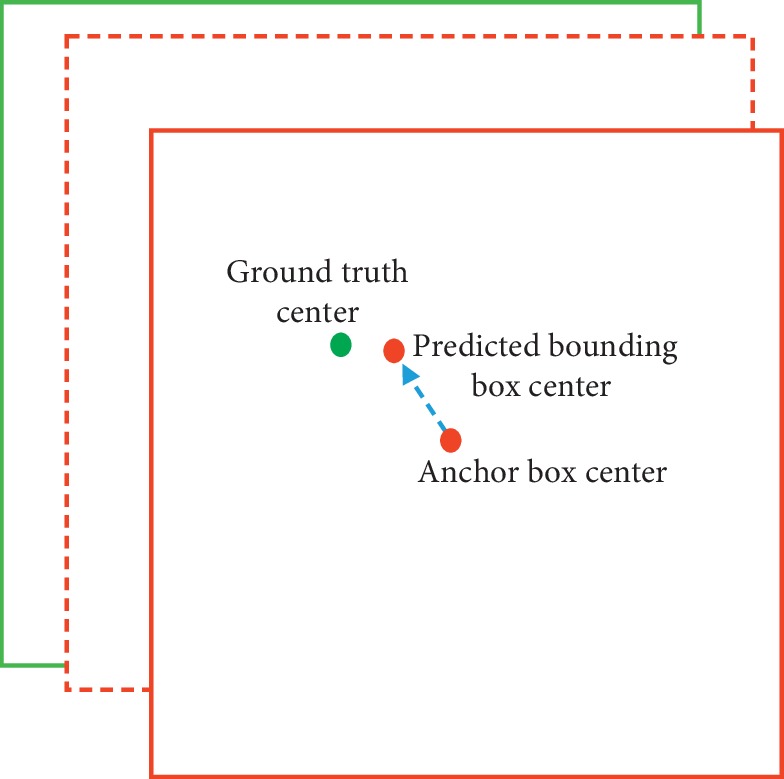
The process of bounding box regression.

In the loss function, *c*_*i*_ is the real category, ${\overset{⌢}{c}}_{i}$ is the prediction category, (*x*_*i*_, *y*_*i*_, *w*_*i*_, *h*_*i*_) is the information of the ground truth, (${\overset{⌢}{x}}_{i}$, ${\overset{⌢}{y}}_{i}$, ${\overset{⌢}{w}}_{i}$, ${\overset{⌢}{h}}_{i}$) is the information of the prediction bounding box, and *λ*_noobj_, *λ*_obj_, *λ*_class_, and *λ*_coord_ are the weight parameters. The MBNet predicts a confidence for each bounding box using logistic regression. The value should be 1 when the anchor box overlaps a ground truth object more than any other anchor box. Its calculation process is shown in equation (10). Unlike YOLOv3 [17], we choose several bounding boxes with the relatively high confidence and average the coordinates of them but do not select only one bounding box with the maximum confidence from highly overlapping detection boxes, as done in [2]. In this way, the localization accuracy for occluded objects is improved, and the recall rate increased by 6.8%. If an anchor box is not responsible for predicting a ground truth object, that is, it does not meet the preset threshold with ground truth box's IOU, it incurs no loss for class predictions, only for confidence predictions, or has a very small weight in the coordinate predictions. The complete bounding box regression process is shown in Figure 7.

(10) $$\begin{array}{c} \text{confidence}=\text{pr}\left(\text{Object}\right)\;*\text{ IOU}\left(\frac{\text{truth}}{\text{pred}}\right), \end{array}$$

(11) $$\begin{array}{c} \text{confidence score}=\text{pr}\left(\frac{{\text{Class}}_{i}}{\text{Object}}\right)\text{pr}\left(\text{Object}\right)\;*\text{ IOU}\left(\frac{\text{truth}}{\text{pred}}\right) \end{array}$$

Besides the 4 coordinates of *b*_*x*_, *b*_*y*_, *b*_*w*_, and *b*_*h*_ and confidence, each bounding box also predicts 7 class scores, corresponding to 7 classes of our handcrafted dataset. By multiplying the confidence value with 7 class scores, respectively, the specific score of the bounding box based on a particular category is then gained, as shown in equation (11). Thus, these confidence scores can be compared to the preset threshold to determine which category should be retained or not. Each bounding box uses multilabel classification to predict the categories a bounding box might contain. For good performance, we do not use softmax but simply use independent logical classifiers: binary cross-entropy loss for the class predictions during the training process. Using a softmax imposes the assumption that each box has only one class, and that is not usually the case; for example, an apple may have labels such as apple, fruit, and food at the same time. The multilabel method can better model the data in the dataset.

## 4. Experiments

### 4.1. Dataset and Evaluation Metrics

A series of comparative experiments are carried out in this paper, we have used a handcrafted urban traffic dataset and the public KITTI dataset to evaluate the performance and effectiveness of the proposed algorithm.

#### 4.1.1. Urban Traffic Dataset

Traffic scenes commonly contain objects (such as all kinds of vehicles and pedestrians) with large-scale variations, as the surveillance cameras usually cover a large and long view of the road. Although publicly available benchmarks have contributed to progress in this area of object detection, existing traffic object datasets often contain a limited range of contents (only cars or pedestrians) and scales, making it difficult to assess real-world performance. In order to demonstrate the proposed method in more practical scenes, we construct a new dataset named urban traffic dataset to provide a better benchmark and focus research effort on these difficult cases.

**Figure 8:**
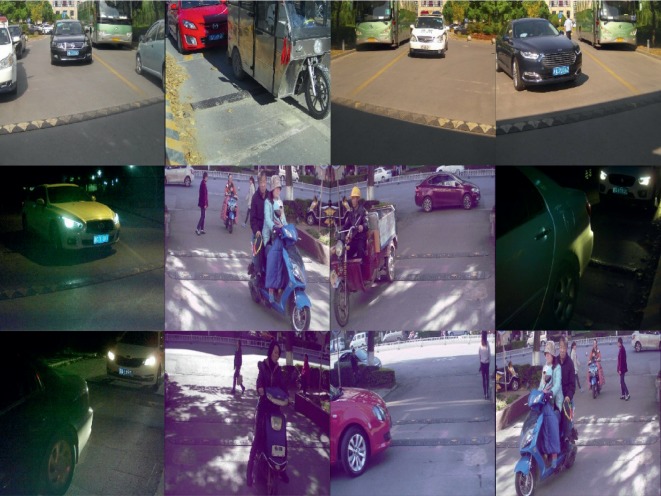
The partial images of urban traffic dataset.

The urban traffic dataset contains objects with a vast variance of scales under traffic scenes, including 10500 well-labeled images under different roads, time, weathers, and traffic states, as shown in Figure 8. The dataset has been divided into three subsets, in which the training set: the testing set: the verification set is 5 : 3 : 2. In detail, it consists 5125 images for training and 3188 images for testing, and verification set is of 2197 images. The dataset consists seven categories, namely, car, car plate, pedestrian, bus, bicycle, motorcycle, and tricycle, which is also the object we need to detect from input images, and it is worth to point out that we treat the car plate as a class for training and testing.

To better fit into the network presented in this paper, we have resized all the images to 512 × 512 resolution. The data distribution of our handcrafted dataset is shown in Table 2. As illustrated in Table 2, the objects are classified into 7 categories under three different scenes (sparse, crowded, and nighttime). We consider a scene as a crowded scene if it contains more than 15 objects per image; otherwise, it is considered as a sparse scene.

#### 4.1.2. KITTI Dataset

KITTI [1] is a widely used benchmark for vehicle detection, which contains objects with different scales in different scenes. The dataset includes 7481 images for network training (including 2494 images for use as a verification set) and 7518 images for testing the model. KITTI dataset provides a 3D border annotation for a moving object captured by using cameras, and the categories of objects include cars, trucks, pedestrians, and bicycles. According to the difference of object size, occlusion, and truncation criteria, the dataset organizer divides the dataset into three levels: easy, moderate, and hard, which can be used to judge the comprehensive performance of various object detection algorithms.

#### 4.1.3. Evaluation Metrics

We employ the universally recognized recall rate, average precision (AP), and intersection over union (IOU) metrics [39] to evaluate the performance of MBNet on our handcrafted dataset, and they have widely been used to evaluate various object detection algorithms [1, 39]. We evaluate the performance of our model for car, pedestrian, bus, bicycle, and so on under the scenes in all cases, such as crowded or sparse and daytime or nighttime. In the experiments, the threshold is set in the range of 0.1 to 0.65, which means that only the overlap between the predicted bounding box and the ground truth greater than or equal to the value will the current detection be considered as a correct detection. Besides, we use the P-R curves and average precision (AP) to present the detection performance of MBNet for cars, cyclists, and pedestrians under scenes with different complexity degree (easy, moderate, and hard) on the KITTI dataset. All the experimental results can be seen in Section 4.4.

### 4.2. Experimental Configuration

Our experiments are implemented on a computer equipped with an Ubuntu 16.04 system and supported by NVIDIA 1060 GPU and Intel(R) Core i7-6700K @ 4.0 GHz∼4.2 GHz CPU. Besides a GPU development package CUDA 8.0 and a deep learning acceleration library cuDNN 6.0 are installed. Then our MBNet is trained under the Python 2.7 environment. The specific parameters of our network are as follows: initial learning rate is 0.001; policy is steps; batch is 64; steps, respectively, take 100, 25000, and 50000; maxbatch is 100000; scales are 10, 0.1, and 0.1; momentum is 0.9, and decay is 0.0005. As shown in Figure 5, the horizontal ordinate represents the number of iterations, ranging from 0 to 100000. After more than 60000 iterations, the parameter has basically been stabilized. During the training process, the change of region average IOU and loss are important parameters to measure the quality of model training, as can be seen from Figures 5 and 6, and the loss is falling and approaching a small constant, while the average IOU is approximately equal to 1, which basically meets the requirements of training.

### 4.3. Explanation of Various Scales

We propose MBNet to effectively detect large, medium, and small objects in the traffic scene, so as to reduce the rate of missing detection. The experiments are carried out on our handcrafted dataset and KITTI, which contains objects with different scales. Through the statistical analysis of the bounding boxes in the dataset, the objects are divided into three categories: small, medium, and large. Specifically, objects with a height or width greater than 10 pixels and smaller than 47 pixels belong to a “small” category; objects with a height or width between 47 pixels and 99 pixels are in a “medium” category. Other objects with a height or width greater than 99 pixels are in the “large” category. The three detection branches of our MBNet can effectively detect these objects from different scenes such as sparse or crowded. The experimental results show that the reasonable design of the detection branch of our model greatly improves the recall rate and the detection precision, and because the MBNet is a 32-layer lightweight network, the speed of processing each image is up to 30 ms (33 fps), which can basically meet the real-time requirements of industry.

### 4.4. Comparison with the State-of-the-Arts

#### 4.4.1. Urban Traffic Dataset

Based on the configuration above, we conduct our experiments drawing support from our premarked dataset, and experimental comparison is made with RCNN, faster RCNN, SSD, mask RCNN, SINet, and YOLOv3, respectively. We make comparative analyses on the recall rate, the average precision, the average IOU, and the time consumption. It is important to note that the YOLOv3 network divides the input images into 13 × 13, 26 × 26, and 52 × 52 small regions (grid cells), and in this paper, we divide the size of the original images into 16 × 16, 32 × 32, and 64 × 64 small regions, respectively. To verify the effectiveness of our method, we compare it with other methods under different thresholds, as shown in Tables 3–9.

Because the recall rate, the detection precision, and the IOU value of each model will change under different thresholds, we compare these metrics under different thresholds (0.1∼0.65). As shown in Table 3, we compare the average precision of the 7 frameworks on the testing set under different thresholds. As seen in the table, the average precision of each method increases as the threshold increases, and this is because a smaller threshold may count in some of the incorrect predictions. As shown in Table 3, our model can obtain the highest average precision in most cases under different thresholds. When the threshold is 0.1 (minimum), the average precision of our method can reach 58.25%, which is 2.84% higher than the SINet and 10.51% higher than that of the SSD network; when the threshold is 0.65 (maximum), the precision of our method reaches 83.68%, which is 1.25% higher than the SINet and 9.29% higher than the SSD network. The average precision of our method can reach near to 60% when the threshold is 0.1, which shows that the network structure proposed in this paper is suitable for the prediction of various objects.  Table 4 shows the comparison of recall rate for various methods, and our model basically has the highest recall rate at different thresholds. This shows that our method has a lower miss detection rate and is more suitable for detecting objects with different scales.

Table 5 [Table tab6]–7 makes the statistics of the detection results for seven categories tested at the threshold of 0.5, and from these tables, we can draw the conclusion that our method has the best detection results compared with other methods under different scenes.

IOU (intersection over union) is mainly used for measuring the overlap degree between the predicted bounding box and the ground truth: the higher the value, the more accurate the prediction. The threshold value set in the experiments is actually the calculated IOU value. As shown in Table 8, we compare the average IOU of seven categories for all methods. At the threshold of 0.1, the average IOU of our model reaches 89.25%, which is 3.38% higher than the YOLOv3 network and 16.8% higher than the RCNN. When the threshold is 0.65, the average IOU of our method reaches 61.58%, and this is also the highest IOU value of all methods. Under other thresholds, our model shows a good advantage over other frameworks.  Table 9 analyzes the time complexity (time consumption) of each framework. Because the RCNN is not an end-to-end network, its time consumption is very high, reaching 3.13 s per image. In addition, our time consumption is lower than that of SSD and Mask RCNNs. Finally, compared with the YOLOv3 network, because our network has only 32 layers, although we take a more detailed partition on the original images, the overall time consumption is lower than the YOLOv3 network.

**Figure 9:**
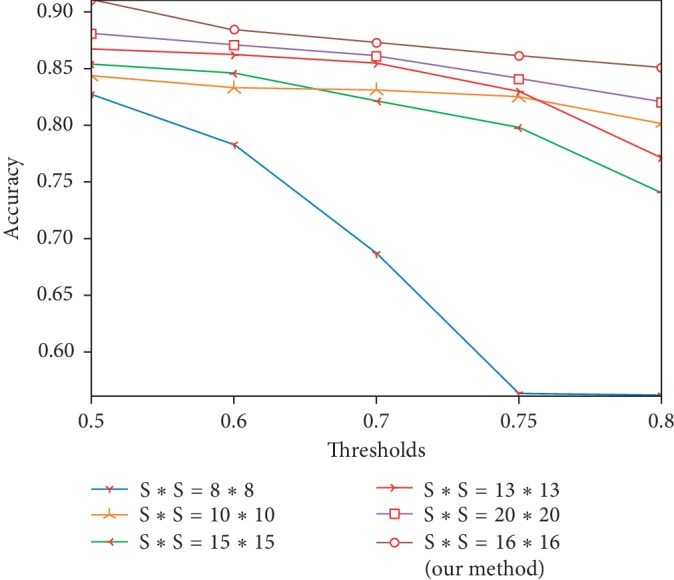
The comparison of accuracy under different partition patterns.

In order to show that the size of 16 × 16, 32 × 32, and 64 × 64 is more adaptable for our model, we have selected five different sizes for comparison, and the results are shown in Figure 9. For the sake of tidiness to demonstrate, each size represents the smallest scale of its group (for example, 16 × 16 stands for 16 × 16, 32 × 32, and 64 × 64). Because each grid cell predicts 3 boxes, the network time consumption will increase with the increase of feature map scale. As seen from Figure 9, when an image is divided into a maximum of 20 × 20 grid, the effect is not as good as 16 × 16 adopted in this paper. In addition, when the input is divided into 8 × 8 grid, the accuracy will decrease rapidly with the increase of threshold, and this is very inappropriate. So the size of 16 × 16 in this paper can be seen as a kind of compromise choice, and its time consumption is not too much, but its accuracy is the best, coupled with our network itself has not many parameters, so in aggregate, the total time consumption is not very high, basically meeting the requirement for real-time performance.

In this section, we compare the recall rate, the average precision, the average IOU, and the time consumption with different methods, and the accuracy under different partition patterns is also discussed. To sum up, our network shows a good advantage over most existing models in the above aspects and can also meet the industry requirements for real-time performance.

**Figure 10:**
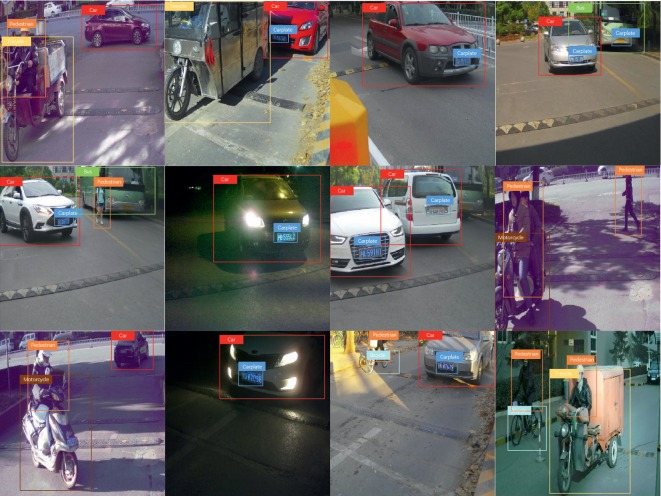
Examples of detection results by MBNet on urban traffic dataset.

In Figure 10, we show some detection results by MBNet on our handcrafted dataset. The results show that the algorithm is effective in detecting objects with different scales, especially for some small-scale objects (such as car plate) in traffic scenes under different conditions such as crowded, sparse, and insufficient illumination. This proves that the proposed MBNet has a good application prospect and has the potential to become an important part of intelligent transportation systems.

#### 4.4.2. KITTI Dataset

**Figure 11:**
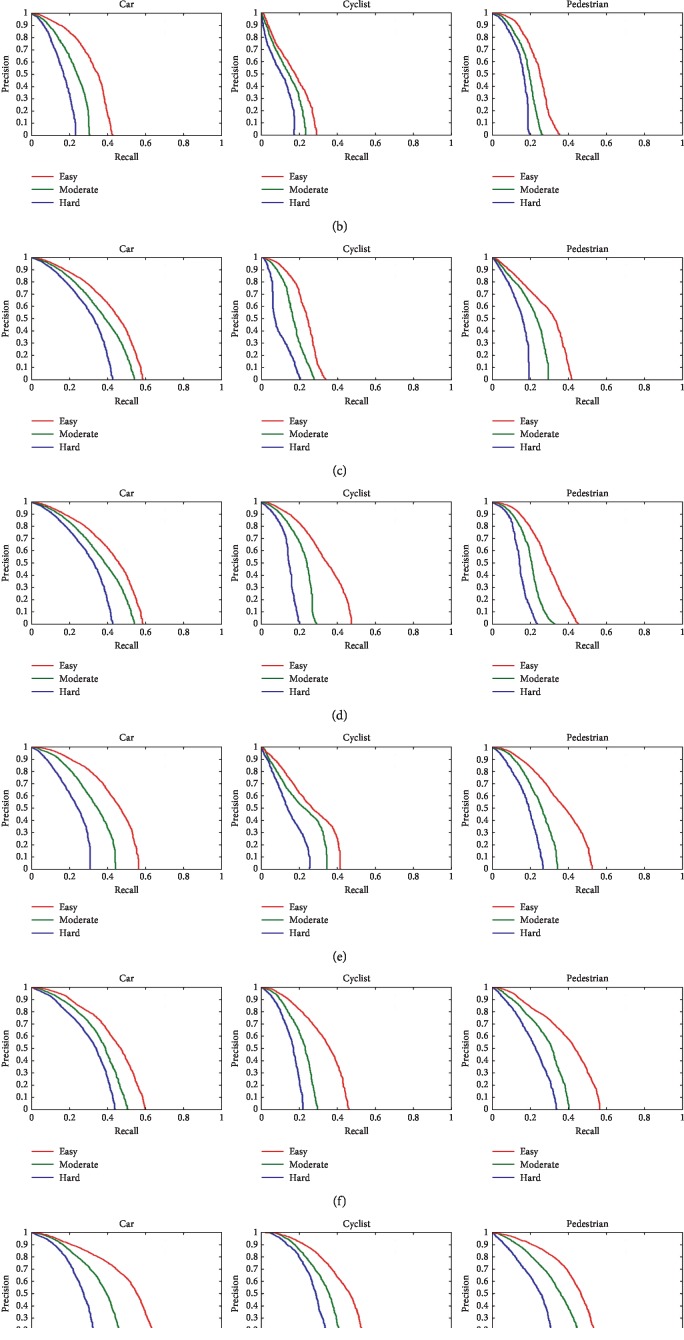
The P-R curves of different objects. (a) RCNN. (b) Faster RCNN. (c) SSD. (d) YOLOv3. (e) Mask RCNN. (f) SINet. (g) Ours.

For further analyzing the effectiveness of the proposed method, we train our model using the KITTI training set and evaluate the model on the testing set of the KITTI benchmark. Specially, we compare the detection performance of RCNN, faster RCNN, SSD, YOLOv3, mask RCNN, SINet, and our method for different objects (cars, cyclists, and pedestrians). The experimental results are shown in Figure 11.

As can be seen from Figure 11, the area under the P-R curves of different objects detected by our method is larger than other methods, that is, the average accuracy of our method is higher, which means the detection performance of our method is better than that of other methods. In addition, we have calculated the average accuracy (AP) for scenes with different complexity degree (easy, moderate, and hard) on KITTI dataset, and the results are shown in Table 10.

**Figure 12:**
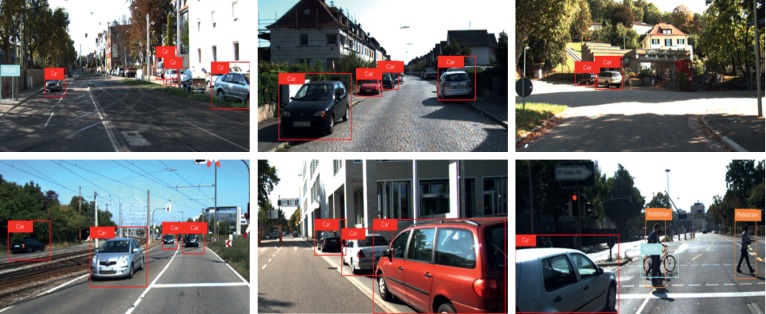
The partial detection results of MBNet on KITTI dataset.

As shown in Table 10, our model can better detect different objects in scenes with different complexity degree, which is due to the reasonable structure design of our model. In Figure 12, we show some of the detection results by MBNet on KITTI dataset. It can be seen from Figure 12 that the network proposed in this paper has a good effect on the detection of vehicles with different scales, which proves the superiority of this algorithm in detecting various objects by using feature maps with different scales.

## 5. Conclusions

To summarize, we propose a 32-layer multibranch network, denoted as MBNet, for fast detection of objects with a large variance of scales in traffic scenes. By designing of three detection branches, it can accurately detect large-, medium-, and small-scale objects from various traffic scenes, such as sparse, crowded, daytime, or nighttime. Besides, we construct a novel labeled dataset, and it contains objects with large-scale variance in traffic scenes, which provides a practical platform for the evaluation of different detection algorithms. The MBNet achieves state-of-the-art performance on both precision and recall rate, and the detection speed is fast enough for real-time detection. The further investigation is to apply MBNet to more challenging datasets as well as have a shot at changing the overall structure of the network for a better performance. What is more, in view of the poor detection effect of most detection algorithms to the dark scene, our follow-up work will also focus on improving the detection effect of the algorithm to scenes with insufficient light.

## Figures and Tables

**Table 1:** The network parameters of MBNet.

Layer	Type	Filters	Size/stride	Input	Output
0	Conv	16	3 ∗ 3/1	512 ∗ 512 ∗ 3	512 ∗ 512 ∗ 16
1	Maxpool		2 ∗ 2/2	512 ∗ 512 ∗ 16	256 ∗ 256 ∗ 16
2	Conv	32	3 ∗ 3/1	256 ∗ 256 ∗ 16	256 ∗ 256 ∗ 32
3	Maxpool		2 ∗ 2/2	256 ∗ 256 ∗ 32	128 ∗ 128 ∗ 32
4	Conv	64	3 ∗ 3/1	128 ∗ 128 ∗ 32	128 ∗ 128 ∗ 64
5	Maxpool		2 ∗ 2/2	128 ∗ 128 ∗ 64	64 ∗ 64 ∗ 64
6	Conv	128	3 ∗ 3/1	64 ∗ 64 ∗ 64	64 ∗ 64 ∗ 128
7	Maxpool		2 ∗ 2/2	64 ∗ 64 ∗ 128	32 ∗ 32 ∗ 128
8	Conv	256	3 ∗ 3/1	32 ∗ 32 ∗ 128	32 ∗ 32 ∗ 256
9	Maxpool		2 ∗ 2/2	32 ∗ 32 ∗ 256	16 ∗ 16 ∗ 256
10	Conv	512	3 ∗ 3/1	16 ∗ 16 ∗ 256	16 ∗ 16 ∗ 512
11	Maxpool		2 ∗ 2/1	16 ∗ 16 ∗ 512	16 ∗ 16 ∗ 512
12	Conv	1024	3 ∗ 3/1	16 ∗ 16 ∗ 512	16 ∗ 16 ∗ 1024
13	Conv	256	1 ∗ 1/1	16 ∗ 16 ∗ 1024	16 ∗ 16 ∗ 256
14	Conv	512	3 ∗ 3/1	16 ∗ 16 ∗ 256	16 ∗ 16 ∗ 512
15	Conv	128	3 ∗ 3/1	16 ∗ 16 ∗ 512	16 ∗ 16 ∗ 128
16	Conv	36	1 ∗ 1/1	16 ∗ 16 ∗ 128	16 ∗ 16 ∗ 36
17	Yolo				
18	Route 14				
19	Conv	128	1 ∗ 1/1	16 ∗ 16 ∗ 512	16 ∗ 16 ∗ 128
20	Upsample		2×	16 ∗ 16 ∗ 128	32 ∗ 32 ∗ 128
21	Route 20 8				
22	Conv	256	3 ∗ 3/1	32 ∗ 32 ∗ 384	32 ∗ 32 ∗ 256
23	Conv	36	1 ∗ 1/1	32 ∗ 32 ∗ 256	32 ∗ 32 ∗ 36
24	Yolo				
25	Route 22				
26	Conv	128	1 ∗ 1/1	32 ∗ 32 ∗ 256	32 ∗ 32 ∗ 128
27	Upsample		2×	32 ∗ 32 ∗ 128	64 ∗ 64 ∗ 128
28	Route 27 6				
29	Conv	512	1 ∗ 1/1	64 ∗ 64 ∗ 256	64 ∗ 64 ∗ 512
30	Conv	36	1 ∗ 1/1	64 ∗ 64 ∗ 512	64 ∗ 64 ∗ 36
31	Yolo				

**Table 2:** The data distribution of our handcrafted dataset.

Scenes	Sparse (daytime)	Crowded (daytime)	Nighttime	Total
Images	7452	1819	1229	10500
Car (C)	37360	14357	8814	60531
Car plate (CP)	37327	14233	8747	60307
Pedestrian (P)	65467	22214	4428	92109
Bus (B)	4216	2456	856	7528
Bicycle (BI)	2213	1710	211	4134
Motorcycle (M)	1104	678	249	2031
Tricycle (T)	2337	742	367	3446

**Table 3:** The comparison of average precision (AP) for various methods (%).

Model	Threshold											
	0.1	0.15	0.2	0.25	0.3	0.35	0.4	0.45	0.5	0.55	0.6	0.65
RCNN [4]	37.32	38.55	40.29	42.78	43.46	44.12	46.29	47.04	51.12	53.47	57.78	59.04
Faster RCNN [9]	41.92	42.47	43.05	45.11	47.38	48.61	50.29	52.33	55.69	59.61	65.29	68.33
SSD [15]	47.74	49.07	52.14	54.97	57.61	60.14	62.24	64.39	65.57	68.14	71.24	74.39
Mask RCNN [13]	51.45	53.23	55.01	56.26	58.38	59.21	62.88	64.49	67.13	70.21	74.44	77.27
SINet [2]	55.41	56.65	57.10	60.21	**65.44**	**68.43**	69.98	72.12	74.56	**79.28**	**81.36**	82.43
YOLOv3 [17]	54.94	55.07	57.34	59.30	62.35	64.82	66.32	68.73	69.18	72.82	75.32	79.73
MBNet	**58.25**	**59.31**	**61.45**	**62.67**	64.94	67.19	**70.01**	**72.33**	**75.12**	77.19	80.01	**83.68**

**Table 4:** The comparison of recall rate for various methods (%).

Model	Threshold											
	0.1	0.15	0.2	0.25	0.3	0.35	0.4	0.45	0.5	0.55	0.6	0.65
RCNN [4]	76.23	74.55	71.29	68.78	65.46	61.12	59.29	57.04	54.35	49.47	46.78	42.04
Faster RCNN [9]	82.92	80.47	78.05	74.11	71.38	68.61	66.29	63.33	61.74	57.61	54.29	50.33
SSD [15]	79.74	77.07	74.14	72.97	70.61	68.14	66.24	63.39	61.27	58.14	55.24	51.39
Mask RCNN [13]	80.65	78.08	76.54	75.15	72.09	68.98	65.66	64.21	63.37	60.25	56.45	53.10
SINet [2]	86.56	85.55	83.03	**82.04**	**78.08**	74.00	70.23	66.49	63.30	61.72	60.28	**59.26**
YOLOv3 [17]	85.94	82.07	80.34	78.30	75.35	73.82	71.32	67.20	**65.14**	61.29	59.32	56.73
MBNet	**88.25**	**86.31**	**83.45**	80.67	77.94	**74.19**	**71.71**	**67.83**	64.20	**62.19**	**60.81**	58.33

**Table 5:** The detection precision for each category under different scenes.

Submeter 1								
Model	Average	Sparse (daytime)						
		*C*	*CP*	*P*	*B*	*BI*	*M*	*T*
RCNN [4]	58.30	58.27	46.16	55.25	67.98	60.66	64.45	55.32
Faster RCNN [9]	63.65	64.93	63.20	66.31	69.29	59.45	60.21	62.17
SSD [15]	71.71	74.25	71.27	69.34	78.78	72.10	66.34	69.91
Mask RCNN [13]	75.85	82.88	68.69	77.71	84.84	69.25	72.12	75.45
SINet [2]	82.20	86.86	74.40	83.66	**87.87**	77.23	**83.25**	**82.10**
YOLOv3 [17]	78.33	84.12	78.27	76.41	84.53	76.37	72.49	76.10
MBNet	**83.79**	**88.63**	**85.52**	**83.98**	86.42	**78.72**	82.71	80.54

**Table 6 tab6:** 

Submeter 2								
Model	Average	Crowded (daytime)						
		*C*	*CP*	*P*	*B*	*BI*	*M*	*T*
RCNN [4]	43.94	42.12	38.28	40.32	55.17	41.31	44.14	46.25
Faster RCNN [9]	47.73	50.25	43.47	51.06	57.28	45.27	44.04	42.74
SSD [15]	58.00	59.27	51.32	62.21	66.57	57.22	58.18	51.21
Mask RCNN [13]	57.78	55.25	58.67	60.20	70.23	56.56	52.33	51.19
SINet [2]	64.95	**66.58**	57.57	**65.35**	73.33	61.01	65.59	65.20
YOLOv3 [17]	60.03	63.63	55.72	58.34	68.63	58.01	56.70	59.21
MBNet	**66.45**	65.78	**61.06**	63.74	**76.59**	**65.70**	**66.81**	**65.46**

**Table 7:** 

Submeter 3								
Model	Average	Nighttime						
		*C*	*CP*	*P*	*B*	*BI*	*M*	*T*
RCNN [4]	10.46	8.84	3.38	11.16	16.55	9.94	10.37	12.95
Faster RCNN [9]	14.71	18.93	9.45	11.17	23.39	8.50	13.22	18.34
SSD [15]	16.15	22.36	11.73	16.66	26.69	9.97	13.35	12.31
Mask RCNN [13]	18.87	24.23	16.66	12.57	28.30	12.29	20.87	17.17
SINet [2]	24.00	30.18	18.24	13.89	**36.21**	**28.35**	24.24	16.87
YOLOv3 [17]	19.47	23.31	12.20	15.57	26.79	20.01	18.11	20.33
MBNet	**27.16**	**33.27**	**21.55**	**18.91**	35.56	27.31	**28.85**	**24.67**

**Table 8:** The comparison of average intersection over union (IOU) for various methods (%).

Model	Threshold											
	0.1	0.15	0.2	0.25	0.3	0.35	0.4	0.45	0.5	0.55	0.6	0.65
RCNN [4]	72.45	71.12	68.67	66.89	63.21	61.72	59.56	56.01	53.29	50.67	46.59	41.21
Faster RCNN [9]	78.23	77.67	76.55	74.31	71.29	69.11	67.47	65.12	62.89	59.61	57.23	54.45
SSD [15]	84.67	82.58	80.69	78.97	76.29	75.17	73.90	70.55	68.22	65.14	62.25	58.30
Mask RCNN [13]	80.34	79.25	78.58	78.10	77.31	75.69	74.26	72.79	70.99	68.68	64.36	60.01
SINet [2]	88.90	**88.08**	82.29	81.81	**80.22**	76.21	73.33	68.68	66.43	64.42	63.99	60.68
YOLOv3 [17]	85.87	84.01	81.44	79.45	77.39	75.88	72.32	69.73	**68.97**	65.82	64.29	61.33
MBNet	**89.25**	87.27	**84.45**	**82.66**	79.99	**77.11**	**74.77**	**71.38**	68.20	**66.23**	**65.01**	**61.58**

**Table 9:** The comparison of time consumption for various methods.

Methods	Time (ms)
RCNN [4]	3130
Faster RCNN [9]	125
SSD [15]	60
Mask RCNN [13]	78
SINet [2]	66
YOLOv3 [17]	40
MBNet	**30**

**Table 10:** The average precision (AP) of different methods on KITTI dataset.

Model	Car			Cyclist			Pedestrian		
	Easy	Moderate	Hard	Easy	Moderate	Hard	Easy	Moderate	Hard
RCNN [4]	44.27	35.49	21.78	30.34	22.17	15.68	41.24	33.55	25.57
Faster RCNN [9]	52.14	41.23	30.77	34.54	25.24	18.29	39.67	26.54	18.23
SSD [15]	83.55	67.87	50.27	57.17	42.14	35.23	62.19	44.53	35.78
YOLOv3 [17]	87.22	71.28	64.67	72.13	60.06	42.77	77.32	65.34	55.58
Mask RCNN [13]	84.39	68.28	58.89	73.68	58.45	40.08	78.32	63.69	50.21
SINet [2]	88.35	**77.49**	62.57	**75.72**	60.29	43.12	80.49	65.97	54.68
MBNet	**88.67**	74.44	**65.98**	74.53	**62.65**	**45.30**	**82.59**	**66.22**	**56.21**

## Data Availability

The data used to support the findings of this study are available from the corresponding author upon request.
